# Antipredator behaviour in semi-feral horses: innate response and the influence of external factors

**DOI:** 10.1007/s10071-025-01933-6

**Published:** 2025-02-04

**Authors:** Antoine Bercy, Francisco Ceacero, Martina Komárková

**Affiliations:** Faculty of Tropical AgriSciences, Czech University of Life Sciences Prague, Kamýcká 129, 165 00 Prague, Czech Republic

**Keywords:** Alert response, Audio-playback, Canids, Equids, Prey, White sound

## Abstract

Rewilding can play a vital role in safeguarding biodiversity, with the grazing pressure exerted by large ungulates and controlled by their predators being a significant factor, particularly in European contexts. Domestic horses are becoming integral to such ungulates’ biomass, but they may differ from truly wild species due to their domesticated origin. This raises concerns about whether feral horses retain adequate antipredator behaviours, especially in the presence of expanding, large predators like wolves. The field of antipredator behaviour research is hampered by inconsistent results and a lack of standardisation, and the behaviour of free-ranging horses remains underexplored. To address this gap, we conducted a playback experiment on semi-feral Exmoor ponies (*n* = 97) in the Czech Republic, exposing them to wolf howls, deer rut calls, and static noise as a control. We assessed alert behaviour and herd grouping while accounting for variables such as herd size, sex, time of day, weather conditions, environment type, presence of other ungulates, and habituation effects. Over 70% of the ponies exhibited alert behaviour in response to both wolf and deer calls. Although the magnitude of responses did not differ significantly between wolf and deer calls, both elicited distinct reactions compared to the control. Most of the studied external factors significantly affected the observed alert responses, highlighting that they must be carefully considered in such studies since these may explain the conflicting results observed in previous studies. The significant behavioural differences in reaction to the sounds indicate that the horses can differentiate them and likely still possess some innate memory, as reported in other ungulates. This is a positive sign towards reintroduction. Future research should carefully consider the validity of the testing environment, habituation effects, and other external factors to ensure robust results.

## Introduction

Natural grasslands have seen a rapid decline and biodiversity loss across Europe while turning to secondary woodlands. A mechanism that may counteract such a turnover is grazing pressure induced by large ungulates and regulated by their predators (Garrido et al. 2019; Ripple et al. 2014). Formerly domestic horses are becoming an integral part of such ungulates’ biomass, but they share a crucial difference as they have been exposed to artificial selection, which may disrupt their innate functional responses to predation risk. Whether antipredator behaviour is driven by learned experience and cognition or by innate genetic mechanisms remains unknown (e.g. Carthey and Blumstein 2018; Janczarek et al. 2020). Natural selection promotes mechanisms to favour antipredator behaviour (Apfelbach et al. 2005), and it is known that hunting success is directly linked to prey vigilance (Quinn and Cresswell 2004). Rewilding efforts also include the resurgence of large predators, such as the wolf, currently observed across Europe (Chapron et al. 2014; Marucco et al. 2023). Recently it was reported that introduced feral equids (*Equus asinus*) in the wild may respond effectively to predation pressure by cougars (*Puma concolor*) due to changes in their activity rate and timing (Lundgren et al. 2022), however, it still remains to be determined if domestic horses living freely or in semi-feral conditions still possess such antipredator reflexes and can adapt to predation, as naïve animals confronted with sudden predation pressure can lead to over-predation issues (Berger et al. 2001).

In antipredator research, numerous variables, including behaviour, physiology, and demography, can influence the results. However, the rationale behind the selection or exclusion of specific metrics is often unclear in many studies. Variations between studies may be attributed to changes in scale rather than ecological shifts (Prugh et al. 2019), a factor that must be carefully considered in behavioural research. Additionally, the risks of habituation and the challenges posed by pseudo-replication (McGregor 2000) can significantly impact findings. To our knowledge, no horse antipredator behavioural study has addressed these issues.

Various methods have been employed to assess horse antipredator behaviour, yielding inconsistent results, largely due to the lack of standardization across studies (Janczarek et al. 2020a, 2021). This inconsistency may also explain why many studies failed to achieve the expected outcomes (Janczarek et al. 2020a). Similarly, a study on Père David’s deer reported mixed results, suggesting that the type of predator—whether pursuit or ambush—is a more significant factor (Li et al. 2011). The uncertainty surrounding the expected responses to predators highlights the limitations of this type of behavioral research, particularly when attempting to compare responses to different predator types.

Two major cues exist for analysing antipredator behaviour: audio cues and olfactory cues. Audio cues have been mostly selected for horses (Janczarek et al. 2020a, b, 2021; Watts et al. 2020) as they were recognised as being a more relevant cue than olfactory ones (Christensen and Rundgren 2008). It has also been observed that interspecific calls from other prey animals can be as significant or even more significant than the predator cue itself (Carrasco and Blumstein 2012; Watts et al. 2020). Next to the predator cue, a neutral sound and an attractive cue are essential. It allows for properly distinguishing the effect of the predator cue compared to the attractive cue (Adcock and Tucker 2020). This 3-stimulus approach allows for better discrimination between them, as a more substantial difference can be expected between the repellent and the attractive than towards the neutral. Anti-predator behaviour is expensive, while the attractive stimulus should reassure the animals and relax their behaviour as less danger is perceived (Dröge et al. 2017).

The first experimental site of this study, the National Nature Monument Mladá, Czech Republic, has seen the introduction of large grazers (Exmoor ponies and backed crossed aurochs) since 2015 to preserve the grassland ecosystem (Dvorský et al. 2022). Exmoor ponies have been used in this effort across the country since then. This brings a unique opportunity to study the antipredator behaviour of ungulates in semi-feral conditions via playback experiments in multiple areas, especially considering the recent return of the wolves to the Czech Republic (Chapron et al. 2014).

This study explores the antipredator behaviour of feral horses, aiming first to determine whether they exhibit any response to potential predators, with the expectation that wolves will elicit the strongest reaction, while deer will provoke a weaker response compared to the control. The second objective is to examine the factors influencing these responses, addressing limitations in previous studies that often yielded inconclusive results due to uncontrolled variables such as sex, herd size, presence of other ungulates, presence of young, weather conditions, and habitat features.

## Methods

### Study sites and subjects

The study was conducted from November 2021 to January 2022. Seven locations across the Czech Republic were used (Table 1). The experiments were conducted on Exmoor ponies (*Equus ferus caballus*) living in semi-feral conditions without veterinary treatment or supplementary feeding, completely undisturbed apart from yearly handling (further information described in Šandlová et al. 2020). All the animals can be individually identified by their distinctive colour patterns and marks. All the ponies used in this study were naïve to wolf howling, but we cannot exclude that some may have heard the deer rut call before. Three different sites comprised females and four other males. The total amount of ponies in the study was 97 (68 females and 29 males). The age of the ponies ranged from 2 to 21 years. The number of ponies was proportionate to the enclosure size (see Table 1) and evaluated to provide adequate grazing. The selected sites had open vegetation with a moderate but variable density of bushes. All the enclosures were fenced, and the entrance was prohibited without authorisation, but walking paths border them. This means there was a frequent presence of humans around the ponies, which were habituated to it. Moreover, they were also habituated to the presence of humans inside their enclosure, as technicians and researchers frequently pass for maintenance and observations. The Milovice enclosure was shared with backed-crossed Aurochs (*Bos taurus*), and the Traviny enclosure was shared with European Bisons (*Bison bonasus*).

**Table 1:** Study sites and herd composition

Sites	Herd composition	Enclosure size (ha)
Milovice	13 mares + 7 young mares	107
Traviny	21 mares + 18 fillies	240
Mašovice	9 mares	28.5
Havraníky	1 stallion + 7 colts	35
Baroch	4 young stallions (no breeding experience) + 8 colts	22.8
Plachta	3 young stallions (no breeding experience)	17.7
Dobřany	6 young stallions (no breeding experience)	32.45

### Playback experiment

This experiment used three sound types: wolf howl as predator sound, deer rut call as interspecific call representing loud, potentially frightening sound, however, produced by non-dangerous herbivores, and white noise as control. To minimise the risk of pseudoreplication (McGregor 2000), it was decided to use six variants for the wolves’ howls and two for the deer calls. The wolf howls come from captive wolves (*Canis lupus baileyi*) at the Wolf Conservation Centre, New York. The deer calls are rut calls from red deer (*Cervus elaphus elaphus* and *Cervus elaphus hippelaphus*) obtained from bioacoustica.org. Based on the availability and suitability of the calls (without disturbing elements), the Mexican wolves instead of the European were chosen; however, as howling is a social communication process that is significant for all canid species (Kershenbaum et al. 2016), it is relevant to use the non-native wolf subspecies for the experiment. The white noise is a static sound generated through the software Avisoft-SASLab Pro (Avisoft Bioacoustics, Berlin, Germany). The sounds were equalised in intensity and length (30 s). The Python *random.choice* command randomly selected sounds for every test.

The sounds were played with a Lamax PartyBoomBox 500 (Lamax, Prague, Czech Republic) at a sound pressure of 100 DB at 1 m, at a distance of 75 m [40DB] measured with a Nikon Monarch 3000 rangefinder (Nikon, Tokyo, Japan). The speaker was covered by dark cloth to decrease its visibility by the horses. This setting avoided overstimulation with an unrealistic sound level while still being hearable by the ponies. At longer ranges, the sound would quickly drop below 30DB and could easily be covered by the sound of the wind.

As the conditions allowed it, it was decided to favour the number of herds over the number of repetitions to avoid any risk of habituation. Every herd was tested only once per sound, and the observations at each herd were carried out with at least one day in between two playbacks, following similar previous studies (Christensen and Rundgren 2008; Janczarek et al. 2020a, b, 2021; Watts et al. 2020). The testing was performed by two people, one playing the sounds and one video recording the herd. The procedure was as follows: the herd was recorded for 5 min without disturbance, then the sound was played for 30 s, and finally, the herd was recorded for another 15 min (Watts et al. 2020). The recordings were performed with a Panasonic HC-VX1 4 K camera (Panasonic, Osaka, Japan). Since remaining hidden was not feasible in most cases, the observers chose not to conceal themselves. The ponies were, however, habituated to human presence and showed no detectable reaction to it.

Other recorded parameters were the herd size, sex and age distribution, with ponies under three 3-years-old considered immature (Rogers et al. 2021). External factors were also noted: the time of the day (hours and minutes), the weather conditions (wind and rain scored as a binary option for the presence or not), which deeply affect horses´ behaviour (Bernátková et al. 2022), the environment type (open or closed habitat) and the presence of other ungulates in the enclosure.

### Data processing

The recordings were analysed following a similar approach as previous studies (Janczarek et al. 2020a, b; Watts et al. 2020). Information about the following behaviours (Table 2) was selected: Grazing, Resting, Alert, Walking, and Trotting (Janczarek et al. 2020a).

**Table 2:** Ethogram of the behaviours studied during the playback experiments

Typeof behaviour	Description
Grazing	Feeding with grass or head low searching for grass patch
Resting	Standing motionless or lying down
Alert	Standing with a high head position, looking around, ears either fixed in the forward position or frequently changing position
Walking	Slow four-beat gait
Trotting	Medium speed two-beat gait or fast three-beat gait

The behaviour of every pony was extracted from the recordings using the software BORIS (DBios, Torino, Italy). The duration of the different behaviours was summarised according to Table 2 for the pre-playback (1 min until the sound started), during the playback (30 s) and post-playback phases (for 1 min after the sound finished). This procedure was chosen according to previous studies showing significant results using this technique (Janczarek et al. 2021). The relative occurrence of each behaviour for every phase was calculated. Since the trotting and alert behaviour were not normally distributed and zero-skewed, the data was thus transformed into binomial variables (yes/no). The data was then further coded into four categories of increasing intensity: no alertness, alert behaviour with the head not pointing to the sound, alert behaviour with the head pointing to the sound, and fleeing away from the sound. This behavioural reactivity response is further referred to as sound reactivity score (SRS). The most frequent behaviour during the pre-playback phase entered the final database as initial behaviour.

The distribution of the herd members in space, further called grouping, was also noted at the end of every phase. This was done by counting the number of the members of the largest group of ponies staying within two horse body lengths. The obtained number was divided by the total amount of ponies. The grouping was not normally distributed for the post-playback; hence, further testing was done on non-parametric procedures. The final database comprised 166 independent observations.

### Statistical analysis

All the statistical tests were conducted in SPSS 28.0.1.1 (IBM, Armonk, New York, USA), with the significance level set at 0.05. Chi-square tests were used to analyse if there were alert or trotting responses to the acoustic stimuli and if it was different for the three sounds played. This was performed with the use of cross-tabulations between the behaviour variable (alertness or trotting) for every phase (pre-, during-, post-playback) and the sound variables (wolf howl, deer call, white noise).

To analyse the differential response to the sounds and the effects of other variables, generalised linear mixed models (GLMM) were performed. The SRS during and post-playback was used as the dependent variable in the GLMMs. Normal distribution and identity link function were used as model settings. The location and the individual pony ID were used to identify the subjects (data structure), and the location was added as a random effect. As fixed effects, the presence of wind, the presence of rain, the herd size, the presence of young, the presence of other ungulates (all of them coded yes/no), the time, the environment type (grassland/shrubland), the sex (male/female), the main behaviour observed during pre-playback (behaviour-pre: grazing/resting), the sound type (deer call, wolf howl, white noise) and the sound playback order (first/second/third) were added. Pairwise comparisons (least significant difference – LSD) were also computed for the SRS of the three sound types tested. The Akaike Information Criterion was finally used to select the most plausible model.

A related-sample Friedmann’s test was performed for each phase to verify if there was a response to the stimuli in the grouping behaviour. Further testing with a GLMM was then performed on the post-playback grouping variable, as its distribution was significantly different from the pre-phase. Location was used to identify the herds and entered the model as a random effect. As fixed effects, the herd size, time, the sound playback order, the presence of wind, rain, young and other ungulates, the environment type, sex, and the sound type were used. The responses to the sound types were again compared among each other through a pairwise procedure. The Akaike Information Criterion was again used to select the most plausible model.

## Results

### Overall reactivity to the sounds

**Fig. 1:**
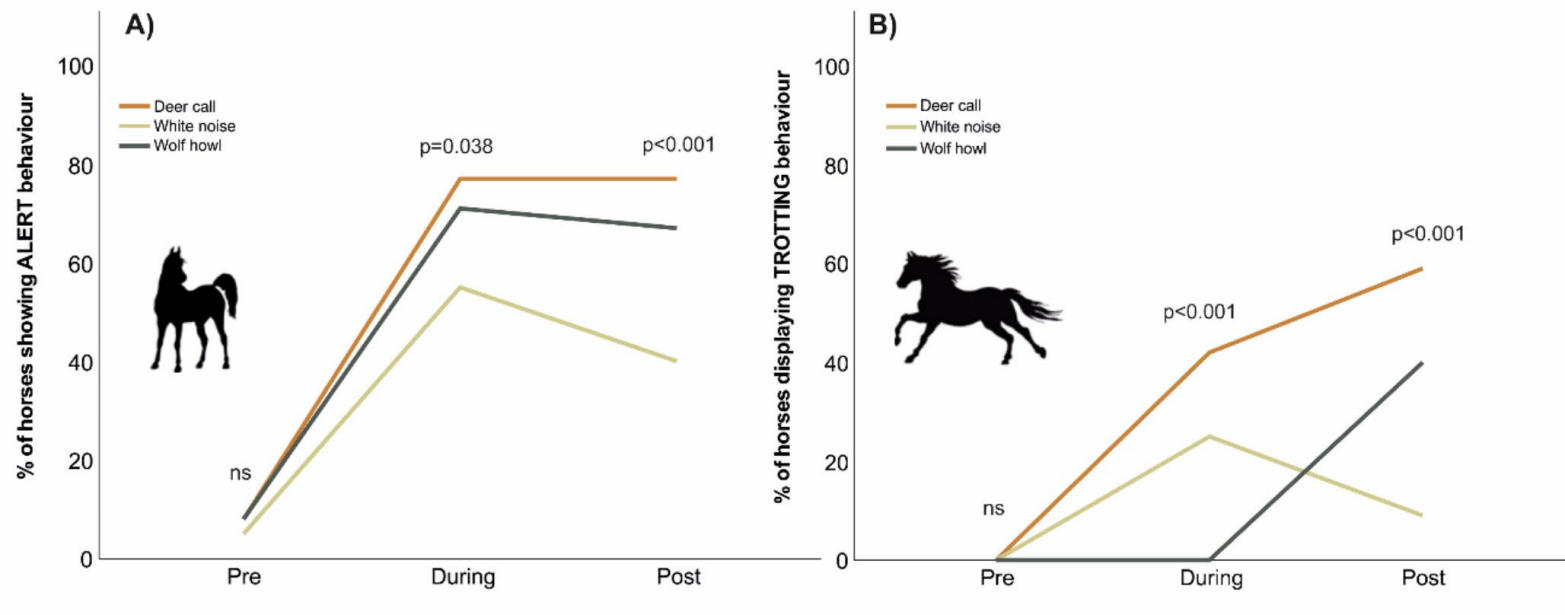
Percentage of horses showing (**A**) alert and (**B**) trotting behaviour along the experiment

The occurrence of alertness was generally low and not significantly different among the sounds during the pre-playback phase (χ^2^ = 0.39; *p* = 0.822), indicating no bias for the subsequent testing. On the contrary, the alertness during- (χ^2^ = 6.54; *p* < 0.001) and post-playback (χ^2^ = 17.52; *p* < 0.001) revealed significant differences among the sounds (Fig. 1A). The deer call elicited the highest percentage of animals displaying alert behaviour, with 77% in both cases (during- and post-playback) reacting to the sound; wolf howls induced 71% and 67% alert behaviour during and post-playback respectively; reactivity to the white noise was the lowest, with 55% during- and 40% post-playback.

Similar results were obtained for the trotting behaviour (Fig. 1B). No analysis could be performed for trotting pre-playback as no animal performed the behaviour during this phase. Trotting behaviour showed significant differences among the sounds both during- (χ^2^ = 24.86; *p* < 0.001) and post-playback (χ^2^ = 31.34; *p* < 0.001). The deer call provoked the highest trotting behaviour, with 42% and 59% of the ponies trotting during- and post-playback, respectively. No trotting was observed during playback for the wolf howl, but it reached 25% for the white sound. On the contrary, post-playback trotting reached up to 40% for the wolf howl but just 9% for the white noise.

### Factors influencing reactivity score during playback

The presence of young, rain, pre-playback behaviour, and time were removed to reach the model with the lowest AIC value. The variables left in the final model (Table 3) were all significant. They included herd size, presence of other ungulates, wind, type of environment, sex, sound playback order and type of sound. Larger herds had stronger reactions towards the sounds (β = 0.176). Herds with other ungulates (β=-1.987) and under windy conditions (β=-0.839) showed a reduced score. It was also lower in open environments (β=-1.097), and females were more reactive than males (β = 1.254). With all these variables under control, the only significant difference in the ponies’ reactivity due to the type of sound was between the deer call and the wolf howl, with the ponies showing a stronger reactivity towards the former (β = 0.433). However, a marginally significant difference in behavioural response intensity between deer call and white noise was found.

**Table 3:** Variables affecting the reactivity of the Exmoor ponies **during** the sound playback phase

Model Term	F_(9,156)_	β (95% CI)	t	*p*-value
Herd size	19.183	0.176 (0.097; 0.255)	4.380	< 0.001
Other ungulates (yes vs. no)	12.761	-1.987 (0.888; 3.085)	-3.572	< 0.001
Wind (yes vs. no)	15.108	-0.839 (-1.266; -0,413)	-3.887	< 0.001
Environment (open vs. closed)	10.038	-1.097 (-1.781; -0.413)	-3.168	0.002
Sex (female vs. male)	6.723	1.254 (0.299; 2.208)	2.593	0.010
Playback order^1^	11.978			< 0.001
Sound type	3.187			0.044
deer vs. white		0.306 (-0.003; 0.615)	1.956	0.052
wolf vs. white		-0.128 (-0.448; 0.193)	-0.787	0.432
deer vs. wolf		0.433 (0.077; 0.790)	2.403	0.017

^1^ Pairwise comparisons for the playback order are not shown since this is just a methodological variable that needs to be controlled for in the model

### Factors influencing reactivity score after playback

For the post-playback GLMM, the presence of young, pre-playback behaviour and time were removed to reach the model with the lowest AIC value, even if the selected model included some non-significant variables (Table 4). Similarly to the previous during-playback model, a larger herd of ponies showed a higher SRS (β = 0.232), the presence of wind again reduced the ponies’ reactivity (β=-0.992), the SRS was lower in open environments (β=-0.880), and the presence of other ungulates reduced the reactivity as well (β=-1.292), but this last effect was just marginally significant. Rain also had a marginally significant effect in the post-playback model (β = 0.696), increasing the SRS. Having all these variables under control, the SRS induced by the type of sound showed an opposite pattern as that observed in the during-playback model: both animal sounds caused a significantly stronger reaction than the white noise (β = 0.722 and β = 0.559 for the deer call and the wolf howl, respectively). However, the difference between the two animals’ sounds was not significant.

**Table 4:** Variables affecting the reactivity of the Exmoor ponies **after** the sound playback phase

Model Term	F_(10,155)_	β (95% CI)	t	*p*-value
Herd size	28,790	0.232 (0.146; 0.317)	5.366	< 0.001
Other ungulates (yes vs. no)	3.253	-1.292 (-0.123; 2.708)	-1.804	0.073
Windy (yes vs. no)	15.387	-0.992 (0.492; 1.491)	-3.923	< 0.001
Rainy (yes vs. no)	3.745	0.696 (0.014; 1.407)	1.935	0.055
Environment (open vs. closed)	3.927	-0.880 (-1.757; -0.003)	-1.982	0.049
Sex (female vs. male)	1.735	0.845 (-0.422; 2.111)	1.317	0.190
Playback order^1^	5.323			0.006
Sound type	9.678			< 0.001
deer vs. white		0.722 (0.366; 1.077)	4.007	< 0.001
wolf vs. white		0.559 (0.221; 0.898)	3.263	0.001
deer vs. wolf		0.162 (-0.219; 0.543)	0.841	0.402

^1^ Pairwise comparisons for the playback order are not shown since this is just a methodological variable that needs to be controlled for in the model

### Grouping response to the sounds

The related-samples Friedman’s two-way analysis of variance by ranks showed a significant difference in the distributions between the grouping pre-, during- and post-playback (*p* = 0.003). However, the pairwise comparisons revealed that only the post-playback phase had a different grouping behaviour than the pre-playback, albeit only marginally significant if considering the adjusted significance (Table 5). Hence, only the post-playback phase was further analysed.

**Table 5:** Pairwise comparison of the grouping phase

Grouping	Test Statistic	Std. Error	Std. Test Statistic	Adj. *p*-value^1^
Pre- vs. During-playback	-0.310	0.309	-1.003	0.948
Pre- vs. Post-playback	-0.690	0.309	-2.237	0.076
During- vs. Post-playback	-0.381	0.309	-1.234	0.651

Each row tests the null hypothesis that the distributions of both samples are the same

^1^ Asymptotic significance (2-sided tests) is shown. Significance values have been adjusted using the Bonferroni correction for multiple tests

For the GLMM for the grouping behaviour in the post-playback phase, the best model (lowest AIC value) contained the sound as a significant variable (F = 10.376, *p* = 0.001). The sound variable showed through the pairwise contrast that the grouping response to the deer call and the wolf howl was identical and higher than the white noise, meaning that more horses grouped together for the natural sounds compared to the control (Table 6).

**Table 6:** Pairwise contrast of sounds post-playback

Sound Pairwise Contrasts	Contrast Estimate	t	Adj. *p*-value
deer – wolf	0	0	1
deer – white	0.533	3.945	0.001
wolf – white	0.533	3.945	0.001

## Discussion

We observed the antipredator response in multiple herds of semi-feral ponies. The playback calls induced an evident change in the horses’ behaviour, but contrary to our expectations, the wolf sound did not cause the most robust response, being tied or surpassed by the deer rut call. In addition, the results were significantly influenced by external factors. Thus, they must be addressed when performing such kinds of studies. The grouping behaviour analysis seems a promising indicator as it showed similar results to the traditional approach of individual alertness observation without the influence of external factors.

One potential issue that could explain the lower-than-expected response to the wolf howl is that it is not an indication of attack and, thus, not a sign of immediate danger (Kershenbaum et al. 2016). It is suggested that the horse is a predominantly visual animal (Miller 1995); this could explain why the type of behaviour is different for the wolf, with no trotting observed during the playback of the wolf sound as they try to identify the sound source. Alternatively, this recalls that predicting subjects’ reactions to predator cues is difficult. It was suggested that ambush predators would impact their prey more than stalking predators (Prugh et al. 2019; Li et al. 2011), which could explain the relatively low response to the wolf howl. The deer rut call shows male vitality, pushes back rivals, and protects its territory (Passilongo et al. 2013). Although no studies have been found about interspecific conflict induced by rut behaviour, the solid response to the deer sound suggests that horses might interpret the antagonistic meaning of such a call. These results highlight the importance of interspecific calls in prey behaviour and that prey can learn to interpret them to increase their situational awareness, as suggested (Carrasco and Blumstein 2012). Post playback, the white noise shows a reduced reaction, especially in trotting. It indicates a possible surprise reaction that quickly wears off as horses identify it as not a real threat, as observed before (Christensen and Rundgren 2008). Using an attractant sound instead of an antagonistic rut call could have helped better prove if the horses can interpret the difference in sounds by inducing behaviour relaxation (Adcock and Tucker 2020; Watts et al. 2020).

To look into standardisation and conflicting results issues and improve our results’ accuracy, several external factors were controlled for in the models and, as expected, showed significant influence on the results. This indicates that differences between similar studies could be explained by managing these variables rather than actual behavioural and ecological differences (Prugh et al. 2019). As hypothesised, the sound order, i.e. habituation, affects the horse’s behaviour (Tables 3 and 4) and thus cannot be ignored. Surprisingly, age and daytime didn’t seem to affect the response in this study. In a previous study, a higher presence of maternal protection towards the young horses and a stronger behaviour responsiveness was observed at dawn or dusk (Watts et al. 2020), albeit the horses were free roaming and not naïve to predators, and a better definition of time relative to dawn or dusk might have altered the horses’ behavioural response. Watts et al. (2020) also observed that predators did not significantly influence the protective behaviour of mares toward their foals, except in terms of maintaining distance. Protective responses were more pronounced in dominant stallions. This may explain why the presence of young did not lead to significant changes in our results. Furthermore, as these were not naturally mixed groups, any behavioural changes would not directly reflect on our methodology. Moreover, the wind affected the response of the horses, likely due to a change in hearing quality. Sound loudness should, therefore, carefully ensure data pertinence and avoid overstimulation, a risk already highlighted (Prugh et al. 2019). In this study, the bigger herds reacted more to the playback, contrasting with other results observed in other ungulates where larger groups showed more relaxed behaviour (Olson et al. 2019). Horses are understudied for this characteristic, but this difference can be due to adaptation to different predators that can lead to different social systems (Feh et al. 1994). We suspect that the size of the group increases the chance of a horse showing flee behaviour, with the rest naturally following, as horses are deeply social animals (Hartmann et al. 2012). However, the presence of other ungulates relaxed the behaviour of the horses, as it was observed in other studies where interspecific cues were interpreted (Watts et al. 2020; Carrasco and Blumstein 2012), suggesting that the interspecific relationship might be more complex than simply adding more members for vigilance. On another note, the stronger response from males could be due to a lack of adequate response, where the resources are preferentially allocated towards mating success (Winnie and Creel 2007; Berger and Gompper 1999). Males might also be bolder as they have a protective role towards the females and young (Saltz and Rubenstein 1995). However, these implications stay limited and inconclusive, as we only saw such a difference during the playback. Nevertheless, this indicates that group composition influences behaviour and cannot be ignored; this difference is significant in groups, and this contrast seems particularly true for isolated horses (Górecka-Bruzda et al. 2022). The variability in behavioural response indicates the complicated and understudied effect of external factors but keeping them under control could help explain the inconclusiveness of the literature on antipredator behaviour. Lastly, other factors, such as the presence of the observers, could also influence the behaviour and should also be considered.

It is also important to point out that this type of testing only shows direct response to stimulus, but it might affect the horses in the long term. The fear or predation can change general behaviour, where the prey might avoid specific locations (Kuijper et al. 2014) or reduce feeding in riskier areas (Shrader et al. 2008). Such behaviour would not be observed in a limited controlled scenario. The ecological relevancy of studies in small, captive environments can also be questioned, as the environment significantly affects the behaviour and does not allow for fleeing behaviour. However, it is still relevant in the context of growing rewilding initiatives.

The group configuration was assessed to have a deeper insight into the anti-predator behaviour; it shows promising results similar to the ones observed in traditional behaviour analysis while not showing the same influence by external factors. The group configuration changed, with reduced distance between horses after the playback of the natural sounds compared to the control (Table 5). Such behaviour is in accordance with other studies on group dynamics, where it is interpreted as a strategy for group defense (Creel et al. 2014; Janczarek et al. 2020a). This shows that grouping could be utilised similarly to more traditional approaches. One major advantage of this method is that it is a more objective way of observation, less prone to behavioural misinterpretation, and eliminating the need for subjective classification, as observed in this study. Group configuration assessment could help to improve future behavioural research but requires deeper investigation, particularly regarding the influence of external factors.

## Conclusions

This study aimed to investigate if naïve horses still possess some antipredator behaviour while observing the efficacy of standard behavioural study methodology. The significant behavioural differences between the sounds indicate that the horses can differentiate them and likely still possess some innate memory, as seen in other ungulates (Adcock and Tucker 2020). This is a positive sign towards reintroduction: naive horses should be able to adapt to the presence of predators. However, horses tend to express flee behaviour and preference for specific environments. A suitable environment is thus required to avoid unnecessary stress (Watts et al. 2020). The same is true for further research; researchers need to consider the validity of the testing environment and habituation and external factors. Otherwise, observed differences in behaviour cannot be accurately dissociated. The herd grouping behaviour seems like a promising antipredator behaviour response as it is less impacted by external factors, albeit it needs more precise measurement techniques and further research.

## Data Availability

No datasets were generated or analysed during the current study.
